# Canine Distemper in Endangered Ethiopian Wolves

**DOI:** 10.3201/eid2105.141920

**Published:** 2015-05

**Authors:** Christopher H. Gordon, Ashley C. Banyard, Alo Hussein, M. Karen Laurenson, James R. Malcolm, Jorgelina Marino, Fekede Regassa, Anne-Marie E. Stewart, Anthony R. Fooks, Claudio Sillero-Zubiri

**Affiliations:** Zoological Society of London, London, UK (C.H. Gordon);; University of Oxford, Tubney, UK (C.H. Gordon, J. Marino, A.-M.E. Stewart, C. Sillero-Zubiri);; Animal and Plant Health Agency, New Haw, UK (A.C. Banyard, A.R. Fooks);; Ethiopian Wolf Conservation Programme, Bale Robe, Ethiopia (A. Hussein, J. Marino, A.-M.E. Stewart, C. Sillero-Zubiri);; Frankfurt Zoological Society, Addis Ababa, Ethiopia (M.K. Laurenson);; University of Redlands, Redlands, California, USA (J.R. Malcolm);; Ethiopian Wildlife Conservation Authority, Addis Ababa (F. Regassa);; University of Liverpool, Liverpool, UK (A.R. Fooks)

**Keywords:** canine distemper, morbillivirus, wolves, endangered, Ethiopian wolves, Canis simensis, Bale Mountains National Park, Ethiopia, canid species, conservation, viruses, epizootics, rabies, vaccination

## Abstract

The Ethiopian wolf (*Canis simensis*) is the world’s rarest canid; ≈500 wolves remain. The largest population is found within the Bale Mountains National Park (BMNP) in southeastern Ethiopia, where conservation efforts have demonstrated the negative effect of rabies virus on wolf populations. We describe previously unreported infections with canine distemper virus (CDV) among these wolves during 2005–2006 and 2010. Death rates ranged from 43% to 68% in affected subpopulations and were higher for subadult than adult wolves (83%–87% vs. 34%–39%). The 2010 CDV outbreak started 20 months after a rabies outbreak, before the population had fully recovered, and led to the eradication of several focal packs in BMNP’s Web Valley. The combined effect of rabies and CDV increases the chance of pack extinction, exacerbating the typically slow recovery of wolf populations, and represents a key extinction threat to populations of this highly endangered carnivore.

## Abstract 2

Investigation into mortalities within endangered species can direct conservation efforts.

Infectious diseases are a major cause of population declines in wildlife (*1*). Canine distemper virus (CDV; family *Paramyxoviridae*, genus *Morbillivirus*) constitutes one such threat and has caused outbreaks in a diverse range of wild mammals: black-backed jackals (*Canis mesomelas*) (*2*); lions (*Panthera leo*) (*3*); spotted hyenas (*Crocuta crocuta*) (*4*); fennecs (*Vulpes zerda*); rhesus monkeys (*Macaca mulatta*) (*5*); and aquatic species, including Lake Baikal seals (*Phoca sibirica*) and Caspian seals (*Phoca caspia*) (*6*). CDV has also affected several threatened carnivores, including the world’s most endangered felid, the Iberian lynx (*Lynx pardinus*) (*7*); the Santa Catalina Island fox (*Urocyon littoralis catalinae*) (*8*); and the Amur tiger (*Panthera tigris altaica*) (*9*). Rapidly expanding human populations increase domestic dog contact with wild canids (*10*,*11*), exacerbating the risk for disease transmission (*12*,*13*). CDV infections in different species are serologically indistinguishable due to the existence of a single stereotype of the virus.

The Ethiopian wolf (*Canis simensis*) is recognized as the rarest canid species in the world and as the most threatened carnivore in Africa. Fewer than 500 adult and subadult wolves remain in half a dozen suitable Afroalpine habitat ranges (*14*). The largest population is in the Bale Mountains National Park (BMNP) in southeastern Ethiopia, where wolf populations reach densities of up to 1.4 adults and subadults/km^2^ (*15*). On average, family packs contain 6 adult and subadults (range 2–20) and protect a home range of ≈6 km^2^ (*16*). Such high wolf densities, large packs, and intense social behaviors increase the risks for disease transmission (*17*). As a result of rabies outbreaks during 1991–1992 (*18*), 2003 (*19*), and 2008–2009 (*20*), wolf subpopulations in BNMP were dramatically reduced by 45%–75% .

Serologic evidence for CDV within wolf populations has been reported (*21*); of 30 samples tested during 1989–1992, a total of 9 (30%) were seropositive for CDV. This finding among wild mammal populations shows that survival rates among animals with canine distemper (CD) infection can be high, as most clearly evidenced in populations of rare or threatened species that are likely to be closely monitored by field conservation efforts. Furthermore, it is well established that the virulence of CDV can vary greatly depending on the infecting virus strain, the immunologic competence of the infected host, and the presence of preexisting infections that can be exacerbated by the immunosuppressive effect of infection with a morbillivirus (*22*).

Population viability analyses have been used to predict the effect of epizootics on wolf populations, and the findings suggest that periodic CD epizootics would play a relatively minor role in population persistence, even when modeled together with rabies (*23*,*24*). However, estimated CD-associated death rates in these models were low (15%–20%), and a caveat of the study findings was that the effect on wolf populations should be reassessed if death rates were >40% (*23*). These models predicted that populations could recover from outbreaks of rabies or CDV, but if the interval between disease outbreaks was <30 months, the likelihood of local extinction would be high in the absence of low-coverage parenteral vaccination campaigns (*25*).

To confirm whether CDV poses an extinction threat to Ethiopian wolves, we examined the effect of CDV infection on pack and population dynamics during 2 CD epizootics in BMNP and quantified their effect on the wolf populations. We investigated the source of CD epizootics in village dogs close to the geographic onset of the outbreaks and compared CD-associated deaths between domestic dogs and wild canids.

Animal care and use protocols for the ethical handling of domestic dogs in this study were approved by the Oxford University Zoology Ethical Review Committee (case no. ZERC040905). Animal care and use protocols adhere to the Animals (Scientific Procedures) Act regulations (1986) in the United Kingdom. Furthermore, all animal handling protocols were approved by the Ethiopian Wildlife Conservation Authority.

## Materials and Methods

**Figure 1:**
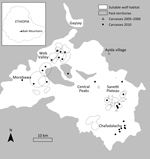
Bale Mountains National Park in Ethiopia, showing location of wolves that died during the 2005–2006 and 2010 canine distemper virus outbreaks in Worgona Valley and Sanetti Plateau and the location of Ayida village, the source of the outbreaks.

The BMNP, in south-central Ethiopia (6°54′N, 39°42′E), contains the largest remaining continuous range of Afroalpine habitat (*26*), upon which Ethiopian wolves are dependent. Wolves in BMNP are found in 3 major subpopulations, all linked by narrow geographic corridors: Morebawa, the Web Valley, and Sanetti Plateau (Figure 1). Wolves are present throughout the Afroalpine range but occur in high densities in these subpopulations.

Since 2001, the Ethiopian Wolf Conservation Programme has closely monitored focal wolf packs in all 3 subpopulations. A total of 18 focal wolf packs were intensively monitored in the 3 areas; 41 packs in other areas, including the Worgona Valley and Chafadalacha, were monitored less intensively (Figure 1) (*27*). Time series of wolf pack densities of the focal packs in Sanetti and the Web Valley were calculated at 6-month intervals; intensive monitoring data and disease information were incorporated in the time series. Wolves up to 1 and 1–2 years of age are classified as juveniles and subadults, respectively; full adult physical appearance and sexual maturity are attained at 2 years of age. We calculated long-term population trends annually and presented them densities of adult and subadult wolves around the time of breeding. To calculate densities, we considered the area occupied by each population as the 95% kernel of all wolf sightings during the breeding season (October–March).

Intensive monitoring enabled the timely recovery of wolf carcasses and the detection of disease epizootics, and detailed pack composition data enabled the recognition of missing wolves, even when their carcasses were not found. Wherever possible, wolf carcasses were subjected to detailed postmortem examination and sampling, following established protocols (*28*). Samples, including lymph node, lungs, spleen, and brain tissue, were collected when possible.

Interviews were conducted in 62 households in Ayida village, 2 km from a wolf pack in Worgona Valley, after reports of a disease outbreak in the area. Domestic dogs that were suspected to have recovered from the virus were captured, and blood samples were obtained for testing.

We analyzed tissue samples for the presence of CDV antibody by using a semiquantitative solid-phase ELISA (ImmunoComb; Biogal, Galed Labs, Galed, Israel). Where possible, we macerated tissues and extracted total cellular RNA by using Trizol (Invitrogen, Carlsbad, CA, USA). Reverse transcription PCR was performed, and a segment of the phosphoprotein (P) gene was generated as described previously (*29*). We sequenced positive reaction products of the correct size (429 bp) in their entirety with primer sequences removed from the consensus. We amplified a section of the hemagglutinin (H) gene in the same manner, using H-specific primers (CDVF1 5′TTAGGGCTCAGGTAGTCCAACA 3′ to CDVR1 5′GACAAGGCCGACTCCAGACAA 3′) to yield a 1,122-bp product. P gene and H gene data were aligned with available data by using MEGA6 (*30*). In all cases, assessing statistical significance using χ^2^ values was done with degrees of freedom = 1.

## Results

### CDV Outbreak 2005–2006

In July 2005, a total of 65 domestic dog deaths were reported in Ayida village (Figure 1), which was just 2 km from the nearest wolf pack in Worgona Valley. In 62 households surveyed, 49% (65/132) dogs owned by villagers had died. Owners commonly reported that infected dogs showed symptoms consistent with a CDV infection, including ocular discharge, convulsive head nodding, loss of appetite, and death. An additional 28% (37/132) of the dogs had been sick but recovered, implying CDV infection in 77% (102/132) of the village dogs. Of 16 serum samples collected from dogs that had recovered, 9 (56%) were positive for CDV antibodies by ELISA.

On September 15, 2005, a wolf with hind leg ataxia, hunching of the back, hair loss, and lethargy was observed in Worgona Valley. On September 21, a wolf carcass was discovered, and in December, a juvenile carcass found. In addition, 7 known wolves disappeared from 4 study packs in the Worgona Valley during September–December. In total, 9 of 19 wolves died or disappeared, resulting in a presumed 47% death rate among adult and subadult wolves across 4 packs.

In November 2005, a known female wolf emigrated from Shiya pack in Worgona to Garba Guracha pack in Sanetti, ≈5 km to the east, and in January 2006, a wolf carcass was discovered in the Garba Guracha pack. During January–April, 13 additional carcasses were recovered in Sanetti (Figure 1), and 10 wolves were observed with clinical symptoms consistent with CDV infection; 4 of the wolves recovered and survived the outbreak. In addition to the 14 wolves that were confirmed dead, 17 other known wolves disappeared from Sanetti during the same period (Table 1), bringing the suspected death rate to 54% (31/58 known wolves) among the 9 packs. Death rates were higher among subadults (83%) than adults (34%). Samples were collected from 3 of 14 carcasses; 2 had positive test results.

**Table 1:** Age and sex distribution of Ethiopian wolves in focal packs monitored in Sanetti Plateau, Ethiopia, before, during, and after a 2005–2006 outbreak of canine distemper virus

Focal pack	November 2005, before the outbreak					April 2006, during the outbreak					November 2006, after the outbreak			
	Adult, M	Adult, F	Subadult	Total		Adult, M	Adult, F	Subadult	Total		Adult, M	Adult, F	Subadult	Total
Badagassa	4	3	3	10		2	2	0	4		2	2	0	4
Batu	3	2	4	9		2	2	0	4		2	2	0	4
BBC	4	1	7	12		4	1	3	8		2	1	0	3
Garba Guracha	2	2	4	8		2	1	1	4		2	1	1	4
Nyala	3	3	2	8		1	1	1	3		1	1	1	3
Quarry	3	2	3	8		3	1	2	6		3	1	2	6
Bilisa	2	1	0	3		2	1	0	3		2	1	0	3
Total	21	14	23	58		16	9	7	32		14	9	4	27

### CDV Outbreak 2010

In April 2010, 3 wolf carcasses were discovered in Web Valley and Morebawa (Figure 1), and during July–August, 9 more were discovered. In September, 5 carcasses were found in Chafadalacha (30 km from Morebawa), and in November, 5 were detected in Sanetti (25 km from Web Valley). In total, 31 carcasses were recovered, and 7 samples were collected for analysis. In addition, 3 domestic dog carcasses were recovered.

In Web Valley, during April–August 2010, a total of 8 wolves were found dead, and another 13 disappeared and were presumed dead, indicating an estimated death rate of 68% (21/31 known wolves from 7 packs) (Table 2). In Sanetti, 8 carcasses were recovered, and 19 more wolves went missing during October–December 2010, indicating a death rate of 43% (27/63 known wolves from 7 packs) (Table 2). In Morebawa, 5 wolf carcasses were found, and 11 wolves went missing during May–August, indicating a death rate of 47% (16/34 from 6 packs). In focal areas, the death rate among subadult wolves (87%, 20/23) was higher than that among adults (39%, 28/71); the death rate among juvenile wolves was 93% (27/29). Ten additional carcasses were recovered in nonfocal wolf areas, such as Chafadalacha and Central Peaks.

**Table 2:** Age and sex distribution of Ethiopian wolves in focal packs monitored in Sanetti Plateau and Web Valley, Ethiopia, before, during, and after a 2010 outbreaks of canine distemper virus

Focal pack	April 2010, before outbreaks					November 2010, during outbreaks					April 2011, after outbreaks			
	Adult, M	Adult, F	Subadult	Total		Adult, M	Adult, F	Subadult	Total		Adult, M	Adult, F	Subadult	Total
Sanetti Plateau														
Badagassa	3	1	3	7		5	2	1	8		3	1	0	4
Batu	4	2	0	6		3	1	0	4		4	1	0	5
BBC	11	3	3	17		7	2	1	10		4	2	1	7
Garba Guracha	4	1	3	8		4	1	2	7		4	1	1	6
Nyala	2	3	2	7		2	1	0	3		1	1	0	2
Quarry	5	3	3	11		4	3	0	7		4	3	0	7
Bilisa	3	2	2	7		3	2	2	7		4	1	0	5
Total	32	15	16	63		28	12	6	46		24	10	2	36
Web Valley														
Darkeena	1	1	0	2		0	0	0	0		0	0	0	0
Mulamu	3	2	1	6		0	0	0	0		0	0	0	0
Meggity	3	1	0	4		2	1	0	3		1	1	0	2
Kotera	1	0	0	1		0	0	0	0		0	0	0	0
Sodota	2	2	6	10		1	1	0	2		0	0	0	0
Alandu	3	2	0	5		3	1	0	4		3	1	1	5
Tarura	2	1	0	3		2	1	0	3		2	1	0	3
Total	15	9	7	31		8	4	0	12		6	3	1	10

For the 2 CDV outbreaks combined, the death rates among subadult (85%) and adult (38%) wolves were significantly higher than the expected annual natural death rate of 15% (*19*) (χ^2^_1_ = 42.98, N = 106, p<0.001) and (χ^2^_1_ = 175.69, N = 46, p<0.001) respectively, but significantly more subadults than adults died or disappeared (χ^2^_1_ = 28.45, N = 152, p<0.001). The death rate among juvenile wolves (93%) during the 2010 CDV outbreak was significantly higher than the expected annual natural death of 37% (*15*) (χ^2^_1_ = 39.16, N = 29, p<0.001).

### CD Diagnosis

**Figure 2:**
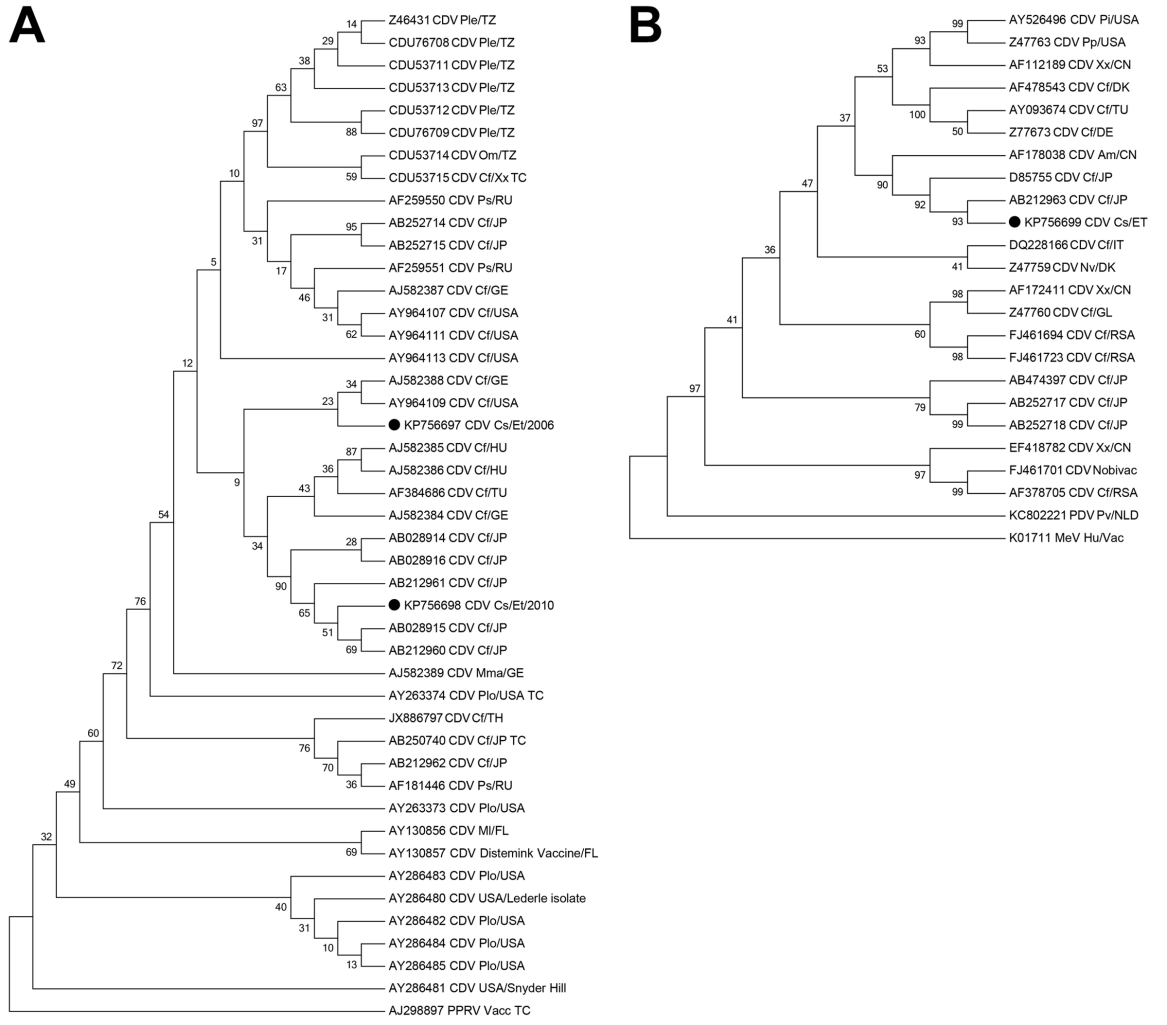
Phylogenetic neighbor-joining trees of canine distemper virus (CDV) isolates from samples collected during outbreaks in 2006 and 2011 (A) and 2010 (B). Evolutionary analyses were conducted in MEGA6 (*30*). A) Tree constructed using the phosphoprotein gene (331 nt). Evolutionary distances were computed using the Kimura 2-parameter method and are in the units of the number of base substitutions per site. The analysis involved 45 nt sequences and a total of 331 positions in the final dataset. B) Tree constructed by using the hemagglutinin gene (1,334 nt). Bootstrap values (10,000 replicates) are indicated at relevant nodes. Black dot indicates Ethiopian wolf samples. Species from which the viruses were isolated are indicated by the following abbreviations: Am, *Ailuropoda melanoleuca* (giant panda); Cf, *Canis familiaris* (dog); Cs, *Canis simensis* (Ethiopian wolf); Hu, human; Mma, *Martes martes* (European marten); Ml, *Mustela lutreola* (European mink); Mm, *Meles meles* (badger); Nv, *Neovison vison* (American mink); Om, *Otocyon megalotis* (bat-eared fox); Ple, *Panthera leo* (lion); Plo, *Procyon lotor* (raccoon); Pp, *Panthera pardus* (black leopard); Ps, *Phoca sibirica* (Baikal seal); Pv, *Phoca vitulina* (harbor seal); Xx, species unidentified. Country of sample origin are indicated as follows: CN, China; DK, Denmark; ET, Ethiopia; FL, Finland; GE, Germany; GL, Greenland; HU, Hungary; IT, Italy; JP, Japan; NLD, the Netherlands; RU, Russia; RSA, South Africa; TU, Turkey; TZ, Tanzania. TC denotes where isolates have undergone extensive tissue culture passage. Phylogenetic outgroups are indicated as follows: PPRV, peste des petits ruminants virus; PDV, phocine distemper virus; and MeV, measles virus.

An amplicon for the P gene could be amplified from only 1 sample among those analyzed from the 2005–2006 outbreak. This sequence grouped phylogenetically with sequences reported for isolates from domestic dogs in the United States and Germany (Figure 2, panel A). Three postmortem samples from the 2010 outbreak were positive for the CDV P gene or the H gene, and the sequences aligned most closely with isolates from domestic dogs in Japan (Figure 2).

### CDV Effects on Population and Pack Dynamics

**Figure 3:**
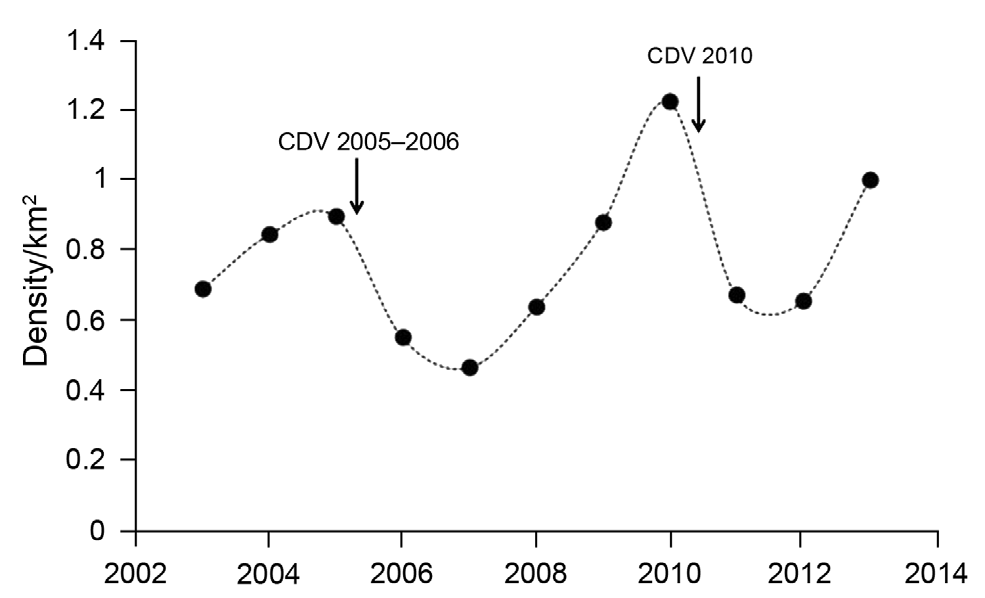
Adult and subadult Ethiopian wolf population in Sanetti Plateau, Ethiopia, 2002–2013. Dots indicate wolf population estimates at different time points; arrows indicate canine distemper virus outbreaks in this study.

Between 2002 and 2013, focal packs in the Sanetti subpopulation were affected by 2 CDV epizootics (2005–2006 and 2010), but no rabies epizootics were observed. Wolf numbers fluctuated in Sanetti in response to CDV infection; the interepizootic interval was 4 years (Figure 3). An immediate lull in population growth followed both epizootics. In 2006, two Sanetti packs (BBC and Lencha) coalesced to form 1 pack, meaning, in essence, that 1 pack became extinct. Breeding success during or immediately after the epizootics was also affected. During 2005–2006, only 4 (44%) focal packs in Sanetti bred; during 2006–2007, only 3 (38%) bred; and during 2010–2011, only 4 (57%) bred. In 2005–2006 in Sanetti, only 4 pups in total survived from 3 packs, but in February during the epizootic, all 4 pups in a fourth pack, Badagassa, died. The remaining 5 packs in Sanetti did not breed that season. Breeding remained suppressed in the 2006–2007 breeding season: only 3 of 8 packs produced pups, of which 10 survived to independence at 6 months of age. During the 2010–2011 breeding season, 3 of 7 focal packs did not breed; another 2 packs bred but lost their pups before emergence at 3 weeks of age. Four of 9 pups from the other 2 packs died before they reached independence.

Subsequent to this initial 2-year lull in reproduction, wolf numbers recovered strongly: by the second outbreak in 2010, wolf numbers and wolf density in Sanetti had surpassed pre-CDV outbreak levels. The combined wolf density for the 7 focal packs in Sanetti more than doubled during 2007–2010 (Figure 3).

**Figure 4:**
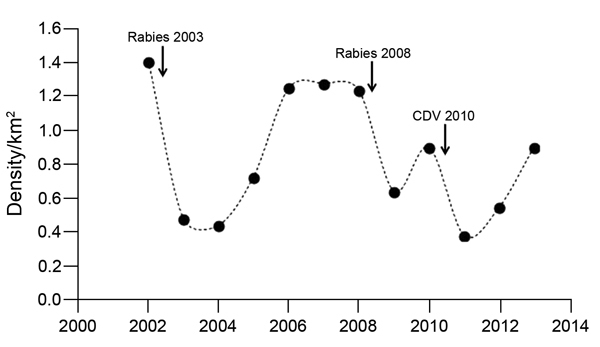
Density of adult and subadult Ethiopian wolf population in Web Valley, Ethiopia, 2002–2013. Dots indicate wolf population estimates at different time points; arrows indicate known rabies epizootics and canine distemper virus outbreaks in this study. Of 7 wolf packs in Web Valley, 4 went extinct after the 2010 canine distemper virus outbreak; in 2011, two new packs formed in the area.

During 2002–2013, Web Valley wolf packs were affected by rabies epizootics in 2003 and 2008–2009 and by an CDV epizootic in 2010 (Figure 4). Death rates were 62% (*19*) and 59% (39/66), respectively, for the 2 rabies epizootics and 68% for the CDV epizootic. Four of 7 Web Valley packs, including 86% of the subadult wolves that were born during the 2008–2009 breeding season, were eradicated by the CDV epizootic. Two new packs formed in the Web Valley in 2012.

## Discussion

### CDV Diagnosis

The detection of CDV-associated deaths among populations of rare Ethiopian wolves is of paramount significance for their effective protection and survival. Alongside the ongoing threat from rabies, deaths from CD highlight the real and present threat that emerging viral diseases pose to these endangered carnivores. Molecular typing of viral pathogens is of great utility in identifying and managing threats to susceptible populations. The phylogenetic clustering of wolf-derived CDV isolates with domestic dog–derived isolates from geographically distinct areas is not surprising. CDV isolates were originally reported to cluster geographically, however, increased reporting and genetic analysis of CDV isolates has shown that translocation of animals, often internationally, can spread the virus globally. Thus, geographically distinct viruses are often found to cluster (*22*). Furthermore, data for CDV isolates are scarce, and our epidemiologic understanding of this virus remains unclear in the absence of genetic data.

### CDV Effects on Population and Pack Dynamics

The detailed death information gathered from both epizootics contradicts the predictions of previous population viability analysis models, which were determined on the basis of lower estimated CDV death rates (15%–20%). Those models indicated that CDV would have little effect on wolf population persistence or pack size (*23*,*24*). However, the observed death rates in our study were 2–4 times higher than those in the earlier studies (*23*). When compared with the effect of natural death rates of ≈15% per year for subadult and adult wolves (*19*), the high death rates observed in our study can rapidly alter population and pack dynamics. The missing subadult and adult wolves in Sanetti in 2005–2006 (29%) and 2010 (35%) and in Web Valley in 2010 (42%) represented more than the 15% natural death rate, providing further confirmation that these missing wolves had died from CD.

Death rates for wolves with CD were comparable to those for dogs in Ayida village. The genetic identity of the virus and the close proximity between the village and wolf habitat makes it almost certain that the village dogs were the source of the 2005–2006 epizootic among wolves; this finding supports the assertion that CDV is transmitted to wild canids by domestic dogs (*12*). Some dogs in Ayida recovered from CD, and the number of surviving dogs combined with the number of dead dogs suggests that the prevalence of CDV infection during the epizootic was much higher than the reported number of deaths. Wolves can also survive exposure to CDV, as evidenced by the detection of a CDV seropositive wolf in 2011 and the detection of seropositive wolves in 1989–1992 (*21*).

Once disease is transmitted from a domestic dog to an individual wolf, the intense social behavior of wolf packs enables pathogens to spread almost instantly within the pack. Adjacent packs interact at territorial boundaries, permitting further transmission through the population. However, the 2 epizootics in this study showed different transmission patterns across subpopulations (Figure 1). The 2005–2006 epizootic spread a relatively short distance from the lower density wolf habitats in Worgona Valley to the adjacent high-density habitats in Sanetti, where the disease died out. In contrast, the 2010 epizootic moved temporally and geographically from Morebawa to Web Valley and Sanetti, crossing through geographic bottlenecks and areas of lower wolf density between these subpopulations. Interpack contact rates are reduced by low pack connectivity within geographic bottlenecks, reducing the probability of disease transmission. This fact reinforces the severity of the 2010 epizootic, which left 64 wolves dead or missing from the 3 focal subpopulations. Monitoring efforts were less intense in the nonfocal, lower density wolf areas, so it was difficult to gauge death in these areas. The reported losses from focal packs, combined with unknown deaths from lower density areas, suggest that this was the single most catastrophic disease event for Ethiopian wolves reported to date; the spread of CDV to all areas of BMNP caused losses that outnumber reports from all previous rabies epizootics (*18*–*20*).

CDV had a considerable effect on younger wolves: death rates among subadults were >2 times higher than those among adults. Lower death rates in adult wolves will aid recovery of packs by keeping breeding units (packs) intact, assuming survival of at least 1 adult female. Although juvenile wolves usually have natural death rates of 37% during high wolf density periods and 29% during periods of population recovery (*15*), juvenile death rates were 3 times these levels after the 2010 epizootic. Lower death rates among adult wolves may reflect previous low-level exposure, and thus immunity.

Although the mechanism by which CDV affects reproduction is uncertain, both CDV outbreaks clearly affected breeding success and pup survival in Sanetti. In periods of high wolf densities, 75% of packs typically breed successfully, and during periods of population recovery, 83% of packs typically breed successfully (*15*); however, <50% of Sanetti packs bred successfully immediately after both CDV epizootics. After this lull, breeding was not impaired, and once the juveniles were recruited into the population, growth rates were rapid in Sanetti: wolf densities doubled over a 3-year period. With the exception of the 2 packs that coalesced, all breeding packs in Sanetti were maintained, and at least 1 adult female survived in each pack. Four years after the 2005–2006 outbreak, wolf densities had recovered above pre-outbreak levels, and signs suggest a similar outcome following the 2010 outbreak.

Ethiopian wolf populations can recover from CDV epizootics, but the capacity to recover will be impaired when intervals between epizootics are short, as was seen in Web Valley. The brevity of the second interepizootic interval (20 months) meant that wolf numbers had only just started to recover following the 2008–2009 rabies epizootic before the CDV epizootic began. After the 2010 CDV outbreak, death rates were high: 4 of 7 packs were eradicated. These pack extinctions confirmed modeling predictions that the probability of pack extinctions greatly increases as the length of the interepizootic period decreases (*23*). Although concurrent rabies and CDV infections likely caused these extreme death scenarios in Web Valley wolves, there is evidence of high death rate CDV epidemics in lions coinciding with high levels of *Babesia* spp. infection resulting from climatic extremes (*31*); thus, other factors should be fully explored.

The loss of breeding units can slow population growth because it is rare for packs of Ethiopian wolves to split (*32*), even though large litter sizes and high juvenile survival may occur following a decrease in population density (*15*,*33*). Dispersal movements are constrained by the scarcity of suitable, unoccupied habitat, although some subordinate females disperse once recruited to adult status (*32*). Packs will expand their territory if opportunities arise, usually following the disappearance of a neighboring pack (*34*). After the 2010 CDV epizootic, available habitat in optimal Afroalpine areas was abundant in Web Valley, and in 2012, two new packs formed (Figure 4), hinting at the resilience of this species. Several of the founder members of these new packs came through the corridor from Morebawa and joined surviving solitary wolves from extinct Web Valley packs. This finding confirms that corridors facilitate migration and recovery after epizootics (*35*), and such migration is critical for minimizing genetic drift from bottlenecks (*36*). Such mechanisms indicate that individual wolves take advantage of breeding opportunities and low population density, filling available habitat to form new packs. The size and structure of the BMNP wolf population lends robustness to wolf numbers and provides greater resilience against disease catastrophes (*23*).

### Conservation Implications

CDV is a major threat to the persistence of some threatened carnivore populations, including the Ethiopian wolves. Long-term disease management plans are vital for conservation of susceptible species, and vaccination of host and target populations remains a key strategy for disease management (*37*,*38*). Even with incomplete CDV control in domestic dogs, any reduction in disease incidence should have a beneficial effect on the persistence of a wild endangered species.

Population viability models indicate that disease-induced population fluctuations and extinction risks can be markedly reduced by the vaccination of a small proportion of wolves (*23*,*25*). However, CDV vaccines for wild species are not currently at the same stage of development as rabies vaccines. In particular, although licensed for domestic species, live attenuated CDV vaccines can cause adverse reactions in wildlife species. Monovalent canarypox-vectored CDV recombinant vaccines hold the greatest promise for protection of wild canids against CDV (*39*), and trials of the Nobivac D and P antigens have also been conducted on wildlife (*40*), with no adverse reactions. The high prevalence of CDV in the surrounding domestic dog population and the apparent frequent incursion of CDV into the BMNP wolves makes finding new disease control strategies all the more urgent, particularly for smaller wolf populations, among which extinction probabilities are even higher with any reduction in interepizootic periods (*23*). The extent of knowledge regarding CDV and its effects is clearly demonstrated in the well-monitored Ethiopian wolf populations, resulting in suggested conservation solutions that are far reaching in their potential application to other susceptible threatened carnivore species.
